# SHoes for Adolescent PatEllofemoral pain: study protocol for the SHAPE Australian community-based, randomised clinical trial

**DOI:** 10.1136/bmjopen-2024-091393

**Published:** 2025-02-07

**Authors:** Kade L Paterson, Sam Shearer, Kim L Bennell, Adam Bryant, Peixuan Li, Anurika P De Silva, Karen Elaine Lamb, Jo-Anne Manski-Nankervis, Rana S Hinman

**Affiliations:** 1 Centre for Health, Exercise and Sports Medicine, Department of Physiotherapy, School of Health Sciences, Faculty of Medicine Dentistry & Health Sciences, The University of Melbourne, Melbourne, VIC, Australia; 2 Centre for Epidemiology and Biostatistics, Melbourne School of Population and Global Health, The University of Melbourne, Melbourne, VIC, Australia; 3 Methods and Implementation Support for Clinical and Health research Hub, Faculty of Medicine, Dentistry and Health Sciences, The University of Melbourne, Melbourne, VIC, Australia; 4 Primary Care and Family Medicine, Lee Kong Chian School of Medicine, Nanyang Technological University, Singapore; 5 Department of General Practice and Primary Care, The University of Melbourne, Melbourne, VIC, Australia

**Keywords:** Adolescent, Knee, Clinical Trial, Musculoskeletal disorders, Child, Chronic Pain

## Abstract

**Introduction:**

Patellofemoral pain affects one-third of adolescents, with most experiencing symptoms into adulthood. Guidelines recommend exercise and foot orthoses based on clinical trials in adults; however, these approaches have been found to be ineffective and have poor adherence in adolescents. ‘Minimalist’ shoes can reduce patellofemoral joint forces in adults; and may therefore be a promising low burden management approach for patellofemoral pain. This article outlines the protocol for a randomised clinical trial (RCT) that aims to determine if minimalist footwear improves pain, function and other symptoms compared with motion control shoes, in adolescents with patellofemoral pain.

**Methods and analysis:**

This is a 3-month, pragmatic, two-arm parallel group, comparative effectiveness, superiority RCT conducted in Melbourne, Australia. We are recruiting 158 participants aged between 12 and 19 years with patellofemoral pain from the community. Following baseline assessment, participants are randomised to receive either minimalist shoes (intervention group) or motion control shoes (control group, given that clinicians typically advocate motion control shoes for patellofemoral pain). Participants choose one pair of shoes in their allocated group from two colour options. They are advised to wear their study shoes for all planned sports and exercise-based activities over the subsequent 3 months and are also advised that they may wear them as much as desired at other times. The primary outcomes are the 3-month change in (1) severity of the worst knee pain experienced over the past week, measured using a Numerical Rating Scale, and (2) the function in sport and play subscale of the Knee Injury and Osteoarthritis Outcome Score for Children. The secondary outcomes include changes in other parameters of knee pain, symptoms, function in daily activities, health-related and knee-related quality of life, global improvement and fear of movement. Other measures include cointervention use, adherence, adverse events, shoe comfort, descriptive characteristics, physical activity levels, footwear characteristics and objective foot measures.

**Ethics and dissemination:**

This study has been approved by the University of Melbourne Greater than Low Risk Human Research Ethics Committee (reference: 2022-25470-35344-4). Written informed consent is obtained from each participant prior to enrolment. The SHoes for Adolescent PatEllofemoral pain trial will provide the first RCT evidence on the efficacy of minimalist shoes compared with motion control shoes in adolescents with patellofemoral pain. Outcomes will be presented at national and international scientific conferences and published in peer-review journals.

**Trial registration number:**

Australian New Zealand Clinical Trials Registry reference: ACTRN12623000042640.

**STRENGTHS AND LIMITATIONS OF THIS STUDY**

- The use of classification criteria to select the study shoes enhances generalisability given clinicians and consumers may use these to recommend any shoes that match the criteria, rather than being restricted to a single shoe make/model.
- We surveyed a large group of adolescents on their preferred shoes (from a selection of eligible models) and colour options for each treatment group, which ensures our shoes are acceptable to adolescents and likely to be worn in the trial.
- While participants will be blinded to the alternative group via limited disclosure, it is not possible to blind participants to their allocated footwear.
- The use of a self-reported measure of adherence may be considered a limitation; however, we will also assess shoe wear time using a temperature microsensor worn inside the study shoes.

## Introduction

 Pain in the front of the knee, termed ‘patellofemoral pain’, affects one-third of adolescents^1^ and is the most common pain site in this population.^2^ The pain is typically aggravated by running, jumping and squatting,^3^ and thus adversely impacts participation in sport and exercise.^4^ Most adolescents (56%) have symptoms 2 years after onset^4^ and nearly half have pain for greater than 5 years and persisting into adulthood.^5^ This poor prognosis persists regardless of treatment,^6^ suggesting current treatments are either ineffective or not used by adolescents.

Clinical guidelines for patellofemoral pain recommend exercise therapy, foot orthoses/insoles and combined interventions (exercise combined with other treatments such as patellofemoral taping or manual therapy).^7^ These recommendations are based on studies in adults given there are only four randomised clinical trials (RCTs) in adolescents.^8^,^11^ While these treatments are effective in adults, they have substantially lower success rates in adolescents (81% vs 38%), even when adherence is similar.^12^ Unfortunately, adherence with these treatments is generally poor in adolescents. In the only RCT of exercise for patellofemoral pain, adolescents completed only 20% of supervised exercise sessions and 36% of home sessions.^9^ Adherence with orthoses is also problematic, with a small feasibility study showing less than half (47%) of adolescents wore their orthoses for the required minimum of 2 hours per day for 5 days per week.^13^ This highlights the urgent need for research on feasible low-burden treatments for patellofemoral pain in adolescents.

Footwear is a promising novel treatment option for adolescents with patellofemoral pain, given specific shoe features can reduce patellofemoral joint forces^14^ that are associated with patellofemoral joint symptoms.^15^ Footwear also overcomes poor adherence associated with current treatments such as exercise and foot orthoses, given most adolescents wear footwear most of the time. Hence, footwear is an inexpensive, easy-to-use approach that may appeal to adolescent’s preferences. Biomechanical research suggests ‘minimalist’ shoes (lightweight shoes with thin, flexible soles) may be beneficial in people with patellofemoral pain. Specifically, running^14 16^ and jumping^17^ in minimalist shoes results in significantly lower patellofemoral joint forces compared with motion control shoes. Given a typical 60 min bout of sport involves ~11 000 steps,^18^ cumulative force reduction with minimalist compared with motion control shoes would be substantial. However, clinicians typically advocate ‘motion control’ footwear (ie, shoes with arch support and cushioned soles) for people with patellofemoral pain,^19^ likely because clinical trials show clinical benefits with inserted foot orthoses that support the medial arch in adults^20^ (although with limited evidence in adolescents^8^). As such, a robust clinical trial comparing the effects of minimalist shoes to motion control shoes in adolescents with patellofemoral pain is warranted.

This study describes the protocol for an RCT that aims to determine if minimalist shoes lead to significantly greater improvements in pain and functional limitations compared with motion control shoes, when worn during planned sport and exercise-based activities over 3 months, in adolescents with patellofemoral pain. The secondary aim of the trial is to determine if minimalist shoes have significantly greater benefits on other clinical outcomes (other parameters of knee pain, symptoms, function in daily activities, health-related and knee-related quality of life, global improvement and physical activity levels) compared with motion control shoes when worn during planned sport and exercise-based activities over 3 months. Given flat flexible shoes have been associated with adverse events in other populations,^21^ research is also needed to evaluate potential adverse events in adolescents with patellofemoral pain.

## Methods and analysis

### Study design

This protocol is described using the Standard Protocol Items: Recommendations for Intervention Trials (SPIRIT; see online supplemental material).^22^ The SHoes for Adolescent PatEllofemoral pain (SHAPE) trial is an individually randomised two-arm, parallel-group, pragmatic, superiority, comparative effectiveness RCT comparing minimalist shoes to motion control shoes conducted in Melbourne, Australia.

### Sample size calculations

Total sample sizes of 126 (63 per arm) and 112 (56 per arm) are required to detect clinically meaningful between-group differences in our pain and function primary outcomes at 3 months, with 90% power and alpha=0.025 (split between the two primary outcomes). For pain, the minimal clinically important difference (MCID) on a Numerical Rating Scale (NRS) is 1.5 units (out of 10).^23^ For function, the MCID on the Knee Injury and Osteoarthritis Outcome Score for Children (KOOS-child) function in sport and play subscale is 10 (out of 100).^24^ The sample size assumes equal SD of 2.38 (extrapolated from pain measured on a visual analogue scale due to a lack of relevant research in adolescent patellofemoral pain)^20^ and 15^24^ for each outcome at 3 months, respectively, for each group. It also conservatively assumes no correlation (intraclass correlation coefficient=0) between baseline and 3 months, given the lack of adolescent footwear RCT data on which to base assumptions. Allowing for a conservative 20% attrition,^21^ we will recruit 79 people per arm (n=158).

### Participants

Participants are being recruited from the community in Melbourne via advertisements with local schools, sporting clubs, social media, our existing sporting networks and our clinical partners. To be eligible, participants must meet the following inclusion criteria:

- Adolescents aged 12–19 years.
- Participate in planned sport and exercise-based activities that load the patellofemoral joint for at least 60 min per week.
- Participate in planned sport and exercise-based activities that load the patellofemoral joint and that cause knee pain either during or after that is ≥3 in severity on an 11-point NRS (0=no pain; 10=worst pain imaginable).
- Report pain in or around the front of the knee that is aggravated by at least one activity that loads the patellofemoral joint during weight-bearing, including squatting, stair climbing, running, hopping and jumping.^7^
- Have a history of knee pain>2 months.

Participants are ineligible for the study if they:

- Are attending primary school (given the different nature of sport and exercise-based activities between primary and secondary schools in Australia).
- Have a history of patellar dislocation.
- Had traumatic onset of knee symptoms.
- Had recent knee surgery in the past 6 months or planned surgery in next 3 months.
- Are unable/not permitted to wear allocated footwear during sport and exercise activities.
- Currently use foot orthoses.
- Were diagnosed in the past 12 months by a health professional as having a knee condition other than patellofemoral pain.
- Self-report other muscle or joint condition anywhere in the body which is worse than study patellofemoral pain.
- Self-report any systemic or inflammatory joint disease (eg, juvenile idiopathic arthritis).
- Have any neurological condition affecting the spine or either lower limb.
- Do not understand written/spoken English.
- Cannot commit to study requirements (eg, only play a sport, or another reason, that precludes wearing of allocated study shoes; attending appointments; completing outcomes; do not have foot size in the range of US 7–13 for men and US 6–11 for women).

### Procedure

**Figure 1:**
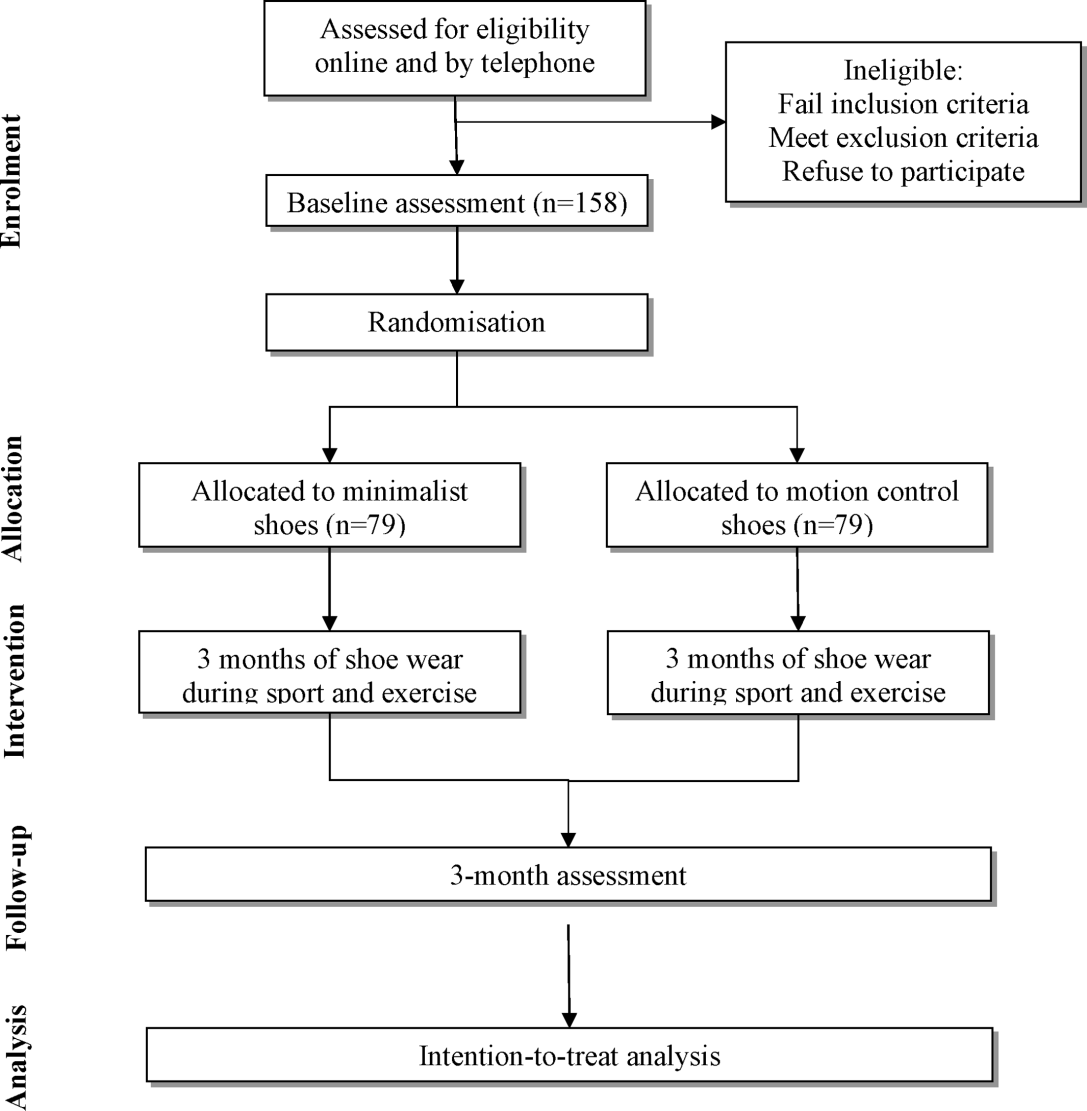
Flow diagram of study phases.

The flow of participants through the RCT is outlined in figure 1. Volunteers are initially screened by an online form, and then over the phone by research staff. This involves a detailed verbal description of the project to ensure that participants/guardians are happy to comply with trial procedures. If participants pass the telephone screening process, participants/guardians are sent the Plain Language Statement (PLS) and consent form in the post or by email (see online supplemental material). After reading the PLS, and if the participant (if aged 16 years or older) or guardian (if the participant is aged under 16 years) gives their consent to participate, they are booked for a baseline laboratory visit at the University of Melbourne. Participants/guardians sign the consent form and provide this to researchers at the start of this visit. They are encouraged to ask researchers any questions or raise any concerns regarding the contents of the PLS and/or consent form before signing if needed.

At baseline, participant-reported outcomes are collected in our laboratory electronically via a tablet computer or on paper with a pen (if preferred). After this, participants are enrolled into the study and randomised to either the minimalist or motion control shoe groups (see below). At the 3-month follow-up, participants are sent questionnaires either on paper or electronically to complete follow-up outcomes at home. A parent/guardian is required to be present when completing all baseline and follow-up outcomes for any participant aged under 16 years.

### Randomisation and blinding

The randomisation schedule was prepared by a biostatistician (1:1 ratio, randomly permuted blocks of varying sizes) and the final randomisation list was generated by an independent statistician who is not involved in the study. The schedule is stored on a password-protected website (REDCap) maintained by a researcher not involved in either participant recruitment or administration of primary/secondary outcome measures. An independent researcher accesses the randomisation schedule to reveal which group a participant is allocated to, after baseline primary/secondary outcomes have been completed.

Participants (and their guardians if aged under 16 years) are blinded to group allocation by a process of limited disclosure. Although participants (also the assessors given participant-reported outcomes) are not blinded to their group allocation, limited disclosure is used to blind them to the alternative condition. Participants/guardians are not informed about the shoe ‘class’ (ie, minimalist or motion control) or models provided to the comparison group participants and are only told that the trial compares two footwear styles that have the potential to improve their kneecap symptoms. Participants/guardians are not informed about the study hypotheses until the completion of the study, at which time they are provided a lay summary of the study purpose, hypotheses and findings. The statistical analysis plan will be written and published on our centre’s website while statisticians are blinded to group allocation. Statistical analyses will be performed blinded to treatment name. Baseline descriptive characteristics that are measured using objective methods in the laboratory (eg, height, weight, foot posture) are measured by an unblinded researcher who also allocates participants to footwear group and fits participants with allocated shoes.

### Footwear interventions

To choose the shoes in each intervention arm, we first reviewed published biomechanical studies which showed minimalist shoes reduce patellofemoral joint contact forces during running and jumping,^14 17^ and other research suggesting motion control shoes may have positive effects on patellofemoral pain.^19^ Key criteria that distinguished minimalist from motion control shoes in these studies matched those we previously used to distinguish flat flexible from stable supportive shoes. These were based on the ability of flat flexible shoes to reduce a proxy measure of tibiofemoral joint force, in adults with knee osteoarthritis^25^ (table 1). We then tested minimalist and motion control shoes matching these criteria during running in 51 young women aged between 17 and 24 years. Our findings (unpublished data) confirmed that the minimalist shoe led to lower patellofemoral joint contact force and stress during a brief bout of running, compared with motion control shoes.

**Table 1:** Shoe characteristics distinguishing minimalist shoes from motion control shoes

	Motion control shoes	Minimalist shoes
Heel height/thickness	>30 mm	<15 mm
Shoe pitch	>10 mm	<10 mm
Arch support/motion control	Present	Absent
Sole flexibility	‘Rigid’ (Footwear Assessment Tool)^41^	‘Minimal’ rigidity (Footwear Assessment Tool)^41^
Weight*	>300 g	≤200 g

Measurements are based on a size 9 US Mmen’s and size 9 US Wwomen’s shoe.

* A tolerance of ±10% was used for shoe weight.

As patellofemoral pain symptoms are aggravated by tasks such as running, jumping and squatting,^3^ footwear has the greatest potential to mitigate symptoms when worn during planned sport and exercise-based activities. Thus, we used our footwear criteria to select a range of commercially available athletic minimalist and motion control shoes suitable for such activities. We then surveyed 677 adolescents aged between 12 and 19 (72% female, 22% male, 3% non-binary, 1% prefer not to say) to ascertain their opinion of the various shoe options and to advise us on their preferred shoe make and model, and two preferred colour options, for each intervention group. The preferred minimalist shoes were the Lems Primal Zen, with preferred colours of black (women’s and men’s) and white (women’s and men’s). The preferred motion control shoes were the Asics Kayano, with preferred colours of black (women’s and men’s), white/blue (women’s) and grey/black/white (men’s).

After group allocation is revealed, participants are asked to choose one pair from the two colour options available in their allocated treatment group. This was to allow for individual preferences and maximise adherence to wearing allocated footwear as instructed. Shoes are fitted by a trained research assistant. Participants are asked to wear their allocated shoes for all planned sport and exercise-based activities over the subsequent 3 months and are also advised that they may wear them as much as desired at other times.

### Outcome measures

Table 2 outlines the enrolment, interventions and outcome measures for this RCT according to SPIRIT recommendations.^22^ A parent/guardian is required to be present when completing baseline and follow-up outcomes for any participant aged under 16 years.

**Table 2:** Schedule of enrolment, interventions and assessments

	Study period					
	Enrolment	Allocation	Post allocation			Close-out
Timepoint	−t_1_	0	1 month	2 months	3 months	3 months
Enrolment						
Eligibility screen	**X**					
Informed consent	**X**					
Allocation		**X**				
Interventions						
Minimalist shoes		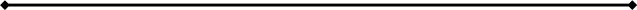				
Motion control shoes		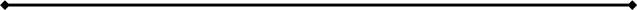				
Assessments						
Primary outcome						
Severity of knee pain (11-point Numerical Rating Scale)		**X**				**X**
Function (KOOS-child function in sport and play subscale)		**X**				**X**
Secondary outcomes						
Achievement of a global improvement in pain						**X**
Knee pain (KOOS-child pain subscale)		**X**				**X**
Knee symptoms (KOOS-child symptoms subscale)		**X**				**X**
Function in daily activities (KOOS-child difficulty with daily activities subscale)		**X**				**X**
Quality of life (KOOS-child knee-related quality of life subscale)		**X**				**X**
Achievement of a clinically relevant improvement in knee pain and function						**X**
Kinesiophobia (Tampa Scale of Kinesiophobia)		**X**				**X**
Other outcomes						
Physical activity (Physical Activity Questionnaire for Adolescents)		**X**				**X**
Shoe wear time			**X**	**X**	**X**	**X**
Adherence to wearing shoes			**X**	**X**	**X**	
Cointerventions		**X**				**X**
Adverse events						**X**
Number of participants who stopped wearing the study shoes						**X**
Comfort						**X**
Treatment expectation		**X**				
Descriptive measures		**X**				
Current planned sport and exercise-based activity participation		**X**	**X**	**X**		**X**
Usual footwear characteristics		**X**				

KOOS-child, Knee Injury and Osteoarthritis Outcome Score for Children

#### Primary outcomes

In chronic musculoskeletal pain, patient-reported outcomes are considered most important for testing treatment effectiveness. Pain and physical function are (1) recommended to evaluate treatment and improve reporting for patellofemoral pain in the latest international patellofemoral pain consensus statement^3^ and by the International Patellofemoral Research Network,^26^ (2) recommended by the Cochrane musculoskeletal group to allow combining/comparing data between trials and (3) widely used in leading international patellofemoral pain clinical trials.^9 20 27^ Pain and physical function are measured at baseline and 3 months, and the primary outcomes are change in pain and physical function, defined as baseline minus follow-up. We will consider minimalist shoes to be more effective if either of the primary outcomes shows benefits.

##### Change in severity of knee pain

The severity of the worst knee pain experienced over the last week is assessed using an 11-point NRS. Scores range from 0 (no pain) to 10 (worst pain possible). The scale has been used as the primary outcome in Cochrane systematic reviews on non-drug treatments for patellofemoral pain,^28 29^ is reliable and valid in adolescents with acute and chronic pain,^30^ and for computer^31^ and tablet-based^32^ use.

##### Change in the KOOS-child function in sport and play subscale

The KOOS-child function in sport and play subscale contains seven questions about function with sport and play activities in the last week.^33^ There are five Likert response options ranging from none (score=0) to extreme (score=5). Scores are normalised to range between 0 and 100, where lower scores indicate worse function in sport and play. The scale has demonstrated good to excellent psychometric properties in children and adolescents, including reliability and validity.^33^

### Secondary outcomes

Secondary outcomes are measured at baseline and 3 months unless indicated otherwise.

#### Achievement of global improvement in pain

Achievement of global improvement in pain is measured at 3 months. Participants rate their overall global change in knee pain since baseline via a 7-point Likert scale, with response options ranging from ‘much worse’ to ‘much better’ when compared with baseline.^34^ Participants that indicate they are ‘moderately better’ or ‘much better’ will be classified as improved. All other respondents will be classified as not improved.

#### Change in the KOOS-child pain subscale

The KOOS-child pain subscale contains eight questions about knee pain experienced in the last week, with five Likert response options ranging from none (score=0) to extreme (score=4). Scores are normalised to range from 0 to 100, where lower scores indicate worse knee pain.

#### Change in the KOOS-child symptoms subscale

The KOOS-child symptoms subscale contains seven questions about knee symptoms in the last week, with five Likert response options ranging from never/none (score=0) to always/extreme (score=4). Scores are normalised to range from 0 to 100, where lower scores indicate worse knee symptoms.

#### Change in the KOOS-child difficulty with daily activities subscale

The KOOS-child difficulty with daily activities subscale contains 11 questions regarding difficulty with daily activities in the last week, with five Likert response options ranging from none (score=0) to extreme (score=4). Scores are normalised to range from 0 to 100, where lower scores indicate greater difficulty with daily activities.

#### Change in the KOOS-child knee-related quality of life subscale

The KOOS-child knee-related quality of life subscale contains six questions about knee-related quality of life experienced in the last week, with five Likert response options ranging from none (score=0) to extreme (score=4). Scores are normalised to range from 0 to 100, where lower scores indicate poorer quality of life.

#### Achievement of a clinically-relevant improvement in knee pain and function

Classified based on individual change in (1) severity of the worst knee pain experienced over the last week and (2) KOOS-child function in sport and play subscale (primary outcomes), where anyone achieving a pain reduction greater than or equal to the MCID of 1.5 NRS units, or 10-unit increase out of 100 on the KOOS-child function in sport and play subscale, is classified as achieving a clinically-relevant pain or function improvement.

#### Change in Tampa Scale of Kinesiophobia

The Tampa Scale of Kinesiophobia contains 17 questions assessing fear of movement, fear of physical activity and fear avoidance, with four Likert responses range from strongly disagree (score=1) to strongly agree (score=4).^35^ Total score ranges from 17 (no kinesiophobia) to 68 (severe kinesiophobia).

### Other measures

Other measures are assessed at baseline and 3 months unless indicated otherwise.

#### Shoe wear time

Shoe wear time is determined using an Orthotimer temperature microsensor. The sensor detects rapid peak changes above ambient temperature and has been previously been shown to be an accurate method for assessing adherence to wearing foot orthoses.^36^ The sensor is positioned within a small (~15 mm × 10 mm) hole cut in the inferior aspect of the arch region of the shoe insole and secured with rigid strapping tape. Participants then walk while wearing the shoe with the sensor in situ to ensure it is comfortable and cannot be felt, with adjustments made to its positioning if necessary. The microsensor is returned by the participant after 3 months using a prepaid express post box, and total wear time is extracted. The number of minutes spent wearing the study shoes, and the number of minutes spent playing sport or exercising while wearing the study shoes, is also self-reported each day during the last week in months 1, 2 and 3 in monthly logbooks.

#### Self-rated adherence with footwear

Participants self-report how much of the time they wore their study shoes during planned sport and exercise-based activities over the previous week, using a 5-point Likert scale, with response options ranging from ‘not at all’ to ‘all of the time’. Participants will complete this scale on the last day of each month for the first 3 months. Participants that indicate they wore the study shoes ‘all of the time’ or ‘most of the time’ during planned sport and exercise-based activities are classified as adherent. All other respondents are classified as not adherent.

#### Cointervention use

Participants complete a custom-developed table at baseline and 3 months to indicate the use of a range of medications and other treatments for their patellofemoral pain over the prior 3 months. Participants who indicate they have used a medication/supplement at least once per week over the past 3 months will be reported as a user of the relevant medication. Participants who have used any other treatment at least once in the past 3 months will be reported as a user of that treatment.

#### Adverse events

Adverse events are defined as any problem experienced in the study knee or elsewhere in the body deemed by the participant to be a result of participating in the trial AND at least one of (1) caused increased pain and/or disability for 2 days or more, (2) resulted in the participant seeking treatment from a health professional and/or (3) caused the participant to miss one or more planned sports or activity sessions. Adverse events are self-reported by participants at 3 months using a custom-developed table. Participants will also be advised that if they experience an adverse event, they can contact the study researchers at any time. A member of the research team (KLP, a study podiatrist) will manage any adverse events reported by participants.

#### Number of participants who stopped wearing the study shoes

Participants are asked to indicate whether they stopped wearing the allocated shoes during the study at the 3-month follow-up visit on a categorical scale (yes or no). Participants who indicate ‘yes’ are asked to describe when and why they ceased wearing their allocated shoes.

#### Comfort

Participants are asked to rate their level of comfort with wearing the allocated shoes during sport/exercise using an 11-point NRS (where 0=extremely uncomfortable and 10=extremely comfortable) at 3 months.

#### Expectation of treatment outcome

Expectation of treatment outcome is rated immediately post randomisation/shoe allocation using a 5-point Likert scale, with anchors of ‘no effect at all’ to ‘complete recovery’.

#### Physical activity

The Physical Activity Questionnaire for Adolescents^37^ contains eight questions about how many days and hours per week adolescents have performed physical activities over the prior 7 days, scored using different 5-point scales. The final score is the mean of each score from the eight items. The score ranges from 1 to 5, with a higher score indicating a higher level of activity.

#### Current planned sport and exercise-based activity participation

Current planned sport and exercise-based activity participation is determined from a predetermined list of sport and exercise-based activities. Participants select which activities they are currently participating in, and how many hours per week they participate in each activity. This is self-reported in the baseline and 3-month follow-up questionnaires and in the last week of months 1 and 2 monthly logbooks.

#### Descriptive measures

Descriptive measures are recorded at baseline and include height, weight, body mass index, age, sex, gender, ethnicity, duration of symptoms, comorbidities (using the Paediatric Comorbidity Index),^38^ foot posture (using the Foot Posture Index, higher scores indicate a more pronated foot)^39^ and current ‘usual’ footwear characteristics (shoe weight, heel height, pitch, arch support and flexibility).^25^

### Statistical analyses

All analyses will be performed by study biostatisticians and will be described a priori in a statistical analysis plan that will be developed while blinded to group allocation and published on our website before analyses commence. Main comparative analyses between groups will be performed using intention-to-treat including all randomised participants according to their allocated study arm. Multiple imputation will be used to account for missing data if the proportion of missing data for either primary outcome is >5%. For the primary outcomes, differences in mean change in pain and function will be compared between groups using linear regression modelling adjusted for baseline values. The primary hypothesis will be evaluated by obtaining the estimated differences between groups in mean change in pain and function from baseline to 3 months, and multiplicity adjusted two-sided 95% CIs and p values. The trial will be considered successful if either of the primary outcomes shows statistically significant (ie, p<0.025) benefits of minimalist shoes over motion control shoes. Findings will also be discussed with regard to the MCID in pain (1.5 NRS units) and function (10-unit increase out of 100 on the KOOS-child function in sport and play subscale). Similar analyses will be conducted for continuous secondary outcomes. For binary secondary outcomes, groups will be compared using risk ratios and risk differences, obtained using log-binomial regression models. Similar analyses will be conducted to compare footwear-related adverse events. To assess whether the effect of the intervention on the primary outcome of NRS pain is moderated by the prespecified moderators of foot posture or participation in jumping based sports, an interaction term between randomised group and moderator will be included in separate models. Additional sensitivity analyses may be conducted and will be specified a priori in the statistical analysis plan.

### Patient and public involvement

We surveyed 677 adolescents aged between 12 and 19 years to help us choose the shoes for each treatment group and to advise on the two preferred colour options. This was to ensure our shoes were acceptable to adolescents and likely to be worn in the trial. We also convened an adolescent stakeholder panel (n=16; one male and one female at each age between 12 and 19 years) to confirm the footwear choices were acceptable, and to provide feedback on the footwear and other study protocols, to review all study materials, to advise on recruiting adolescents and to advise on dissemination of findings at the completion of the trial.

### Trial status

We prospectively registered our RCT on the 16 January 2023 (ACTRN12623000042640). Participant recruitment began in March 2023 and is expected to be completed by June 2026.

### Ethics and dissemination

This study has been approved by the University of Melbourne Greater than Low Risk Human Research Ethics Committee, reference number 2022-25470-35344-4. Written informed consent is being obtained by all participants prior to enrolment. Outcomes will be presented at national and international scientific conferences and published in peer-review journals. Our consumers and clinical partners will help us prepare a dissemination plan targeted to adolescents and clinical end users.

## Discussion

The SHAPE trial will be the first RCT to compare the efficacy of minimalist shoes to motion control shoes in adolescents with patellofemoral pain. We hypothesise that minimalist shoes, worn during all planned sports and exercise-based activities over 3 months, will be more effective than motion control shoes, for improving knee pain and function in sport and play. We also hypothesise that minimalist shoes will be more effective in improving secondary outcomes than motion control shoes. Biomechanical studies show that minimalist shoes reduce patellofemoral joint contact forces associated with knee pain in adults,^14 16 17^ supporting their potential to have clinical benefits. However, the effects of minimalist shoes on pain and function have not been investigated in adolescents. Although a recent feasibility RCT found greater improvements in knee pain in adolescents who wore minimalist-style school shoes to those wearing traditional school shoes,^40^ this study was not powered to compare efficacy and participants continued to wear their regular athletic shoes (which contained an ‘elevated heel’) for sports and exercise activities during the trial.

Adolescents nearly always wear footwear for sporting and other exercise-related activities that exacerbate knee pain, thus athletic footwear represents a novel low-burden treatment option to reduce pain and improve function. Unlike adults, there are no clinical guidelines for managing adolescents with patellofemoral pain. Findings from the SHAPE RCT will provide the first evidence regarding the effects of footwear for managing adolescents with this painful condition. The outcomes of this trial may be used to inform future clinical guidelines for adolescents with patellofemoral pain, improving the clinical management of this important but neglected patient group. Our findings may also be used as an impetus for footwear research in adults with patellofemoral pain or other musculoskeletal pain conditions.

## supplementary material

10.1136/bmjopen-2024-091393 online supplemental file 1
