# Supplementary material for: SHoes for Adolescent PatEllofemoral pain: study protocol for the SHAPE Australian community-based, randomised clinical trial
Source: BMJ Open. 2025 Feb 7;15(2):e091393. doi: 10.1136/bmjopen-2024-091393 (PMC11815409; doi:10.1136/bmjopen-2024-091393)
Supplement: online supplemental file 1 [file bmjopen-15-2-s001.docx]

SHoes for Adolescent PatEllofemoral pain: study protocol for the SHAPE randomised, community-based clinical trial

Supplementary materials

SHAPE Consent form over 15 years Page 2

SHAPE consent form under 16 years Page 4

# Consent Form

## Centre for Health, Exercise and Sports Medicine

## Department of Physiotherapy

## **Project:** SHoes for Adolescent PatEllofemoral pain: the SHAPE randomised clinical trial

**Responsible Researcher:** Dr Kade Paterson

Tel: 03 8344 0425 Email: kade.paterson@unimelb.edu.au

**Additional Researchers:**

Prof Rana Hinman, Prof Kim Bennell, Assoc Prof Adam Bryant, Dr Jo-Anne Manski-Nankervis, Dr Karen Lamb, Ms Joanna Ling, Mr Sam Shearer

| **Name of Participant:** |  |
| --- | --- |

1. I consent to participate in this project, the details of which have been explained to me, and I have been provided with a written plain language statement to keep.
2. I understand that the purpose of this research is to investigate the effects of different commercially available footwear styles on kneecap pain and other symptoms.
3. I understand that my participation in this project is for research purposes only.
4. I acknowledge that the possible effects of participating in this research project have been explained to my satisfaction.
5. In this project I will be required to participate in a 90-minute laboratory visit, where I will undergo a running assessment and complete a questionnaire. I will then wear the allocated study shoes during all planned sport and exercise-based activities for 3 months, during which time I will also complete 3 logbooks. Finally, I consent to completing a second questionnaire at the completion of the study.
6. I understand that my participation is voluntary and that I am free to withdraw from this project anytime without explanation or prejudice and to withdraw any unprocessed data that I have provided.
7. I understand that the data from this research will be stored at the University of Melbourne and will be destroyed 15 years after I turn 18.
8. I have been informed that the confidentiality of the information I provide will be safeguarded subject to any legal requirements; my data will be password protected and accessible only by the named researchers.
9. I understand that after I sign and return this consent form, it will be retained by the researcher.
10. I understand that the data from this research may be used in other future research projects.

| **Participant Signature:** |  | **Date:** |  |
| --- | --- | --- | --- |

# Consent Form

## Centre for Health, Exercise and Sports Medicine

## Department of Physiotherapy

## **Project:** SHoes for Adolescent PatEllofemoral pain: the SHAPE randomised clinical trial

**Responsible Researcher:** Dr Kade Paterson

Tel: 03 8344 0425 Email: kade.paterson@unimelb.edu.au

**Additional Researchers:**

Prof Rana Hinman, Prof Kim Bennell, Assoc Prof Adam Bryant, Dr Jo-Anne Manski-Nankervis, Dr Karen Lamb, Ms Joanna Ling, Mr Sam Shearer

| **Name of Participant:** |  |
| --- | --- |

1. I consent to allow my child to participate in this project, the details of which have been explained to me and my child, and I have been provided with a written plain language statement to keep.
2. I understand that the purpose of this research is to investigate the effects of different commercially available footwear styles on kneecap pain and other symptoms.
3. I understand that my child’s participation in this project is for research purposes only.
4. I acknowledge that the possible effects of participating in this research project have been explained to my satisfaction.
5. I acknowledge that in this project my child will be required to participate in a 90-minute laboratory visit that I will attend, where they will undergo a running assessment and complete a questionnaire. They will then wear the allocated study shoes during all planned sport and exercise-based activities for 3 months, during which time they will also complete 3 logbooks with my assistance. Finally, I consent to my child completing a second questionnaire at the completion of the study with my assistance.
6. I understand that my child’s participation is voluntary and that they are free to withdraw from this project anytime without explanation or prejudice and to withdraw any unprocessed data that they have provided.
7. I understand that the data from this research will be stored at the University of Melbourne and will be destroyed 15 years after my child turns 18.
8. I have been informed that the confidentiality of the information my child provides will be safeguarded subject to any legal requirements; my child’s data will be password protected and accessible only by the named researchers.
9. I understand that after I sign and return this consent form, it will be retained by the researcher.
10. I understand that the data from this research may be used in other future research projects.

I, _____________________________________________________ consent, on behalf of

_____________________________________________________, to participate in the above project.

**Parent/Guardian Signature: ______________________________________ Date: _________________**
