# Supplementary material for: SHoes for Adolescent PatEllofemoral pain: study protocol for the SHAPE Australian community-based, randomised clinical trial
Source: BMJ Open. 2025 Feb 7;15(2):e091393. doi: 10.1136/bmjopen-2024-091393 (PMC11815409; doi:10.1136/bmjopen-2024-091393)
Supplement: Supplementary file 2 [file bmjopen-2024-091393.reviewer_comments.pdf]

PEER REVIEW HISTORY

BMJ Open publishes all reviews undertaken for accepted manuscripts. Reviewers are asked to complete a checklist review form and are provided with free text boxes to elaborate on their assessment. These free text comments are reproduced below.

ARTICLE DETAILS

Title (Provisional)

SHoes for Adolescent PatEllofemoral pain: study protocol for the SHAPE Australian community-based, randomised clinical trial

Authors

Paterson, Kade L; Shearer, Sam; Bennell, Kim L; Bryant, Adam; Li, Peixuan; De Silva, Anurika P.; Lamb, Karen Elaine; Manski-Nankervis, Jo-Anne; Hinman, Rana S

VERSION 1 - REVIEW

**Reviewer**	**1**
**Name**	**Alonazi, Asma**
**Affiliation**	**Majmaah University, Physical Therapy**
**Date**	**08-Sep-2024**
**COI**	**No Competing interests**

Thank you for submitting your manuscript, Your study addresses an important research question regarding the efficacy of minimalist shoes versus motion control shoes in adolescents with patellofemoral pain. I appreciate the thoroughness with which you have defined the research objectives, outlined the study design, and addressed the ethical considerations.

The abstract is well-balanced and accurately reflects the content of the study. The methods are detailed and should allow for reproducibility, while the statistical analysis plan is appropriate for addressing the research question. I also acknowledge your consideration of potential study limitations and the inclusion of ethical approvals and trial registration.

I look forward to seeing the results of your trial and the contribution it will make to the field of adolescent patellofemoral pain management. Thank you for your efforts and contribution to this area of research.

Here are my comments:

1. Grammar and English Acceptability

o Some sentences are unnecessarily lengthy, which may confuse readers. For example, in the methodology section, there are long sentences that could be split into more digestible parts for easier reading.

o There are minor grammatical errors, such as inconsistent tense usage and awkward phrasing, which could be smoothed for clarity. For instance, "This scale is completed on the final day in months 1, 2, and 3..." could be simplified as "Participants will complete this scale on the last day of each month for the first three months."

o Ensure consistent formatting of references, especially when listing multiple sources in a sentence.

2. Objectives

o The objectives of the study are clearly defined. It aims to compare the effectiveness of minimalist shoes to motion-control shoes in reducing knee pain and improving function in adolescents with patellofemoral pain.

o The manuscript could further emphasize the novelty and importance of the study in the context of existing research. Although it touches on this, expanding on how this study fills a gap in the literature would enhance the justification for the trial.

3. Abstract

o The abstract does not clearly state the primary outcomes and secondary outcomes, which are essential for understanding the study's focus. Adding these will make the abstract more comprehensive.

o Briefly mention the study's significance or the potential impact of the findings in the abstract to give readers a sense of why this research is crucial.

8. Outcomes Clearly Defined

o The secondary outcomes, such as the effect of the intervention on footwear-related adverse events, are mentioned but could be more clearly delineated in the abstract and introduction. A simple summary table could help outline all primary and secondary outcomes for quick reference.

o More clarity is needed on how the success of the trial will be determined—what threshold of pain/function improvement will be considered clinically significant?

4. Clarity

o The overall structure of the manuscript is logical, and each section follows a natural progression.

o The methods section is quite detailed and provides clear descriptions of the intervention and assessments.

o Ensure that every acronym is fully spelled out when first introduced, and consistently used throughout the manuscript.

5. Study Limitations

o The manuscript could better address other limitations. For example, what are the potential biases that could arise from self-reported adherence? How will these be mitigated?

o The long recruitment period could also be a challenge, and factors like potential dropout rates should be mentioned.

6. Discussion

o The rationale for choosing minimalist shoes is well-explained, supported by biomechanical studies that link them to reduced knee pain in adults.

o The discussion could delve deeper into the clinical implications of this research. For example, if successful, how would this research influence current guidelines for adolescent patellofemoral pain management?

o Consider expanding on the potential for broader applications of the findings, beyond adolescents or knee pain specifically.

7. Conclusion

o The conclusion could be more definitive in stating the expected outcomes and their significance to both scientific literature and clinical practice.

**Reviewer**	**2**
**Name**	**Koeter, Sander**
**Affiliation**	**Canisius Wilhelmina Hospital, Department of Orthopedics**
**Date**	**17-Sep-2024**
**COI**	**none**

Feedback article:

Thank you very much for the opportunity to review this article. In this work a study protocol for a randomized clinical trial comparing minimalist footwear to motion control shoes in a two arm parallel group design. Aim is asserting superiority of the intervention over usual care in a group of juvenile (age 12-19 years) participants.

The hypothesis that minimalist shoes lead to greater improvement in pain and functional limitations compared to motion controlled shoes is based on biomechanical research, showing that specific shoe features can reduce patellofemoral joint forces.

Generally, I think the article can have more structure (introduction, methods, results, discussion, conclusion). At the moment, some sections are merged.

Methods and analysis:

Study design is well described.

It is unclear to me on what scientific published or unpublished data sample size is based, please clarify.

Is adjusted for loss to follow up or limited shoe wear time or non-adherence?

non-adherence to the protocol accounted for?

Participants: please clarify if additional therapy (such as physical therapy) is allowed or that intervention is restricted to motion control or minimalist footwear. If additional therapy is allowed, how is that accounted for in the analysis?

Is previous episode of physical therapy for 12 weeks or more an exclusion criteria? Previous research on this subject is available. Perhaps the authors can refer to the PEX trial?

Are specific sport such as football, field hockey, rugby or other pivoting sports excluded? Please specify.

Randomisation

No comments.

Footwear interventions

No comments

Outcome measures

KOOS child function subscale and severity of pain are considered primary outcome. A NRS score is used for pain and a 7 question subscale of the full KOOS is used. Is data on validation for use of this subscale as primary outcome available?

Statistical analysis

No specific comments.

Textual considerations:

Page 3: Last sentence of introduction: "improves pain, function and other symptoms", shouldn't it be "improves function and reduces pain and other symptoms"? Or do you want to see if minimal footwear results in more pain and other symptoms compared to motion control shoes?

On the same page at the primary outcomes, for the second primary outcome, a scale has been given, for the first one there has not been one given. However in the article itself there is a scale.

Page 22: There is no clear section of strenghts and limitations of the study in the discussion section.

Looking at the above, I think some revision needs to be done and some addition information is required. I would like to leave it to up to the editor of this journal to decide how to proceed.

**Reviewer**	**3**
**Name**	**Dack, Kyle**
**Affiliation**	**University of Bristol, MRC Integrative Epidemiology Unit**
**Date**	**10-Nov-2024**
**COI**	**None**

This manuscript describes a protocol to evaluate the effectiveness of treating adolescent knee pain and function using minimalist compared to motion-control shoes.

The protocol is very well written, containing a comprehensive description of the planned methods and statistical analyses, and the rationale behind methodological decisions. The methods and analysis plan appear sound and I have no further suggestions or requests for clarification.

VERSION 1 - AUTHOR RESPONSE

Reviewer: 1

Thank you for submitting your manuscript, Your study addresses an important research question regarding the efficacy of minimalist shoes versus motion control shoes in adolescents with patellofemoral pain. I appreciate the thoroughness with which you have defined the research objectives, outlined the study design, and addressed the ethical considerations.

The abstract is well-balanced and accurately reflects the content of the study. The methods are detailed and should allow for reproducibility, while the statistical analysis plan is appropriate for addressing the research question. I also acknowledge your consideration of potential study limitations and the inclusion of ethical approvals and trial registration.

I look forward to seeing the results of your trial and the contribution it will make to the field of adolescent patellofemoral pain management. Thank you for your efforts and contribution to this area of research.

RESPONSE: Thank you for this positive feedback and for taking the time to review our manuscript.

GRAMMAR AND ENGLISH ACCEPTABILITY

1. Some sentences are unnecessarily lengthy, which may confuse readers. For example, in the methodology section, there are long sentences that could be split into more digestible parts for easier reading.

RESPONSE: In response, we have broken up several of the longer sentences in our Methods section.

2. There are minor grammatical errors, such as inconsistent tense usage and awkward phrasing, which could be smoothed for clarity. For instance, "This scale is completed on the final day in months 1, 2, and 3..." could be simplified as "Participants will complete this scale on the last day of each month for the first three months."

RESPONSE: This sentence has been changed as advised. We have also checked our manuscript to ensure consistency of tense. Please note that our trial is ongoing hence uses current tense, as well as past and future tense, as required.

3. Ensure consistent formatting of references, especially when listing multiple sources in a sentence.

RESPONSE: Reference formatting has been checked to ensure consistency throughout our manuscript and with the journal formatting requirements.

OBJECTIVES

4. The manuscript could further emphasize the novelty and importance of the study in the context of existing research. Although it touches on this, expanding on how this study fills a gap in the literature would enhance the justification for the trial.

RESPONSE: We note that the reviewer has included this comment under their "Objectives" heading. We don't feel it's appropriate to add this information in the objectives, but would be happy to be guided by the Editor if they feel it is warranted. Instead, we have emphasised the novelty throughout our Introduction section, where we have placed our study within the context of existing literature.

For instance, we have highlighted the poor adherence and efficacy of existing treatments for patellofemoral pain in adolescents, and stated that "*This highlights the urgent need for research on feasible low-burden treatments for patellofemoral pain in adolescents*."

In the subsequent paragraph we explain why "*Footwear is a promising novel treatment option for adolescents with patellofemoral pain*" and conclude "*a robust clinical trial comparing the effects of minimalist shoes to motion control shoes in adolescents with patellofemoral pain is warranted*."

ABSTRACT

5. The abstract does not clearly state the primary outcomes and secondary outcomes, which are essential for understanding the study's focus. Adding these will make the abstract more comprehensive.

RESPONSE: The Reviewer may have missed these, which were provided in our Abstract:

"*The primary outcomes are the 3-month change in i) severity of the worst knee pain experienced over the past week, and ii) the function in sport and play subscale of the Knee Injury and Osteoarthritis Outcome Score for children. Secondary outcomes include changes in other parameters of knee pain, symptoms, function in daily activities, health- and knee-related quality of life, global improvement, and fear of movement. Other measures include co-intervention use, adherence, shoe comfort, descriptive characteristics, physical activity levels, footwear characteristics, and objective foot measures*."

6. Briefly mention the study's significance or the potential impact of the findings in the abstract to give readers a sense of why this research is crucial.

RESPONSE: We have added the following to the Ethics and Dissemination section of our Abstract:

"*The SHAPE trial will provide the first RCT evidence on the efficacy of minimalist shoes compared to motion control shoes in adolescents with patellofemoral pain*."

OUTCOMES CLEARLY DEFINED

7. The secondary outcomes, such as the effect of the intervention on footwear-related adverse events, are mentioned but could be more clearly delineated in the abstract and introduction. A simple summary table could help outline all primary and secondary outcomes for quick reference.

RESPONSE: Thank you for picking up that we hadn't included adverse events in our Abstract. We have now added it as follows:

"*Other measures include co-intervention use, adherence, adverse events, shoe comfort, descriptive characteristics, physical activity levels, footwear characteristics, and objective foot measures*."

We have also added the following to our Introduction:

"Given flat flexible shoes have been associated with adverse events in other populations[1], research is also needed to evaluate potential adverse events in adolescents with patellofemoral pain."

Thank you also for the suggestion of a summary table of our outcomes. These were included in "Table 2: Schedule of enrolment, interventions and assessments."

8. More clarity is needed on how the success of the trial will be determined—what threshold of pain/function improvement will be considered clinically significant?

RESPONSE: Firstly, we have explained in the "Primary outcomes" section of our Methods that the primary aim will be supported if either the pain or function outcome is found to be superior:

"*We will consider minimalist shoes to be more effective if either of the primary outcomes shows benefits*."

This will be determined by obtaining the estimated differences between groups in mean change in pain and function from baseline to 3 months, and multiplicity adjusted two-sided 95% confidence intervals and p-values. The trial will be considered successful (i.e., the null hypotheses will be rejected) if either of the primary outcomes shows statistically significant (i.e., $p<0.025$) benefits of minimalist shoes over motion control shoes. When discussing our findings, we will also consider the magnitude of any between group differences relative to the minimal clinically important difference in pain (1.5 numerical rating scale units) and function (10-unit increase out of 100 on the KOOS-child function in sport and play subscale).

Whilst this was explained in the "Statistical analysis" section of our Methods, we have also added the following:

"*The trial will be considered successful if either of the primary outcomes shows statistically significant (i.e., p<0.025) benefits of minimalist shoes over motion control shoes. Findings will also be discussed with regards to the minimal clinically important difference in pain (1.5 numerical rating scale units) and function (10-unit increase out of 100 on the KOOS-child function in sport and play subscale).*"

CLARITY

9. Ensure that every acronym is fully spelled out when first introduced, and consistently used throughout the manuscript.

RESPONSE: We have checked our manuscript and all acronyms have been fully spelt out in the first instance and used consistently.

STUDY LIMITATIONS

10. The manuscript could better address other limitations. For example, what are the potential biases that could arise from self-reported adherence? How will these be mitigated?

RESPONSE: We have added the following to our limitations:

"*The use of a self-reported measure of adherence may be considered a limitation, however we will also assess shoe wear time using a temperature microsensor worn inside the study shoes*."

11. The long recruitment period could also be a challenge, and factors like potential dropout rates should be mentioned.

RESPONSE: Thank you for this comment. Our recruitment period is based on our successful track record of recruiting this population at the same rate (4 participants/month),[2] and we have been fully funded for recruitment during this period, and the remainder of our study. Hence recruitment over this period is unlikely to be a challenge. Our sample size calculation (see Methods) has allowed for 20% attrition similar to our previous footwear trials.[1]

DISCUSSION

12. The discussion could delve deeper into the clinical implications of this research. For example, if successful, how would this research influence current guidelines for adolescent patellofemoral pain management?

RESPONSE: Whilst we included a statement on how our findings may influence clinical guidelines, we have clarified this further as follows:

"*Findings from the SHAPE RCT will provide the first evidence regarding the effects of footwear for managing adolescents with this painful condition. Outcomes of this trial may be used to inform future clinical guidelines for adolescents with patellofemoral pain, to improve the clinical management of this important but neglected patient group*."

13. Consider expanding on the potential for broader applications of the findings, beyond adolescents or knee pain specifically.

RESPONSE: Although a broader application of our findings is speculative and beyond the scope of our trial, we have added the following statement to our conclusion:

"*Outcomes of this trial may be used to inform future clinical guidelines for adolescents with patellofemoral pain, to improve the clinical management of this important but neglected patient group. Our findings may also be used as an impetus for footwear research in adults with patellofemoral pain or other musculoskeletal pain conditions*."

14. The conclusion could be more definitive in stating the expected outcomes and their significance to both scientific literature and clinical practice.

RESPONSE: We have amended our Discussion to further clarify our expected outcomes:

"*We hypothesise that minimalist shoes, worn during all planned sports and exercise-based activities over 3 months, will be more effective than motion control shoes, for improving knee pain and function in sport and play. We also hypothesise that minimalist shoes will be more effective in improving secondary outcomes than motion control shoes*."

Please see our responses to comments 18 and 19 regarding the implications of our findings for clinical practice.

Reviewer: 2
Thank you very much for the opportunity to review this article. In this work a study protocol for a randomized clinical trial comparing minimalist footwear to motion control shoes in a two arm parallel group design. Aim is asserting superiority of the intervention over usual care in a group of juvenile (age 12-19 years) participants.

The hypothesis that minimalist shoes lead to greater improvement in pain and functional limitations compared to motion controlled shoes is based on biomechanical research, showing that specific shoe features can reduce patellofemoral joint forces.

15. Generally, I think the article can have more structure (introduction, methods, results, discussion, conclusion). At the moment, some sections are merged.

RESPONSE: Our original manuscript already included this structure, with the exception of a results section given this is a study protocol and hence does not have any results- thus a results section is not relevant nor appropriate. However, we have now capitalised our main section headings to make this structure clearer: Abstract, Introduction, Methods and Analysis, Discussion and References. These headings are also consistent with BMJ Open's recommended headings. Finally, we have ensured no sections are merged.

Methods and analysis:

16. It is unclear to me on what scientific published or unpublished data sample size is based, please clarify.

RESPONSE: Our sample size is based on detecting a clinically meaningful between-group differences in our pain and function primary outcomes at 3-months, which has been fully explained and referenced in the "Sample size" section of our Methods:

"*For pain, the minimal clinically important difference (MCID) on a numerical rating scale (NRS) is 1.5 units (out of 10).*[3] *For function, the MCID on the Knee Injury and Osteoarthritis Outcome Score for Children (KOOS-child) function in sport and play subscale is 10 (out of 100).*[4] *The sample size assumes equal standard deviations of 2.38 (extrapolated from pain measured on a visual analogue scale due to a lack of relevant research in adolescent patellofemoral pain)*[5] *and 15*[4] *for each outcome at 3-*

months respectively for each group. It also conservatively assumes no correlation (intraclass correlation coefficient=0) between baseline and 3-months given the lack of adolescent footwear RCT data upon which to base assumptions. Allowing for a conservative 20% attrition,[21] *we will recruit 79 people per arm (n=158)*.

(Note: References are reproduced at the end of this response to review)

17. Is adjusted for loss to follow up or limited shoe wear time or non-adherence? non-adherence to the protocol accounted for?

RESPONSE: We are not sure if the Reviewer is asking whether i) the analyses will be adjusted for loss to follow-up, limited shoe wear-time or non-adherence OR ii) if the loss to follow-up of 20% accounts for limited shoe time or non-adherence.

We have assumed that the Reviewer is asking about i). However, please be aware that the 20% attrition in our sample size calculation (see response to #22 above) does not include limited shoe time or non-adherence. The primary analysis will be conducted on an intention-to-treat basis according to the randomised treatment group, irrespective of shoe wear time or non-adherence.

Assuming that the reviewer is asking about how loss to follow-up, limited shoe wear-time or non-adherence are dealt with in the analysis, as specified in the Statistical Analysis section, multiple imputation will be used to account for missing data if the proportion of missing data for either primary outcome is >5%. This approach will account for any missing data due to loss to follow-up.

Data on shoe wear time/non-adherence will be examined and reported descriptively. We may perform sensitivity analyses estimating treatment effects on the primary outcomes assuming full adherence. In this manuscript, we have briefly outlined a plan for our main statistical analyses. Additionally, as specified in the Statistical Analysis section, all analyses, including any additional sensitivity analyses, will be described in detail *a priori* in a statistical analysis plan that we will publish on our centre's website before analyses commence:

"*All analyses will be performed by study biostatisticians and will be described a priori in a statistical analysis plan that will be developed while blinded to group allocation and published on our website before analyses commence*."

We have added a line to explain that we may perform additional sensitivity analyses, as follows:

"*Additional sensitivity analyses may be conducted and will be specified a priori in the statistical analysis plan*."

Participants:

18. please clarify if additional therapy (such as physical therapy) is allowed or that intervention is restricted to motion control or minimalist footwear. If additional therapy is allowed, how is that accounted for in the analysis?

RESPONSE: Other than planned surgery, participants will not be restricted from using other treatments. The primary hypotheses will be assessed by estimating the treatment effects, regardless of any use of additional therapy. Co-intervention use will be measured at baseline and 3 months and the number and proportion of participants using the medication and/or cointervention will be reported. This will be fully outlined in our statistical analysis plan (see response to comment 23 above).

19. Is previous episode of physical therapy for 12 weeks or more an exclusion criteria? Previous research on this subject is available. Perhaps the authors can refer to the PEX trial?

RESPONSE: Previous physical therapy (or any treatment other than surgery) is not one of our exclusion criteria. Whether an adolescent has undergone previous treatment (or not) was not considered applicable because if they pass our inclusion and exclusion criteria, they are still symptomatic and hence may benefit from the footwear intervention, even if they have previously had physical therapy treatment.

20. Are specific sport such as football, field hockey, rugby or other pivoting sports excluded? Please specify.

RESPONSE: We have not excluded specific sports. However, in response to this feedback we have amended our final exclusion criteria (unable to commit to study requirements) to make it clearer that participants who compete in sports such as these, which require sports-specific shoes, will be excluded:

"xii) cannot commit to study requirements (e.g., only play a sport, or another reason, that precludes wearing of allocated study shoes").

We have also not excluded pivoting sports, however our inclusion criteria outlines that they must participate in sports that "*load the patellofemoral joint for at least 60 minutes per week*", "*load the patellofemoral joint and that cause knee pain either during or after*" and "*pain in or around the front of the knee that is aggravated by at least one activity that loads the patellofemoral joint during weightbearing, including squatting, stair climbing, running, hopping, jumping*".

Outcome measures

21. KOOS child function subscale and severity of pain are considered primary outcome. A NRS score is used for pain and a 7 question subscale of the full KOOS is used. Is data on validation for use of this subscale as primary outcome available?

RESPONSE: This information was provided for our pain primary outcome as follows:

"*The scale has been used as the primary outcome in Cochrane systematic reviews on non-drug treatments for patellofemoral pain,*[6 7] *is reliable and valid in adolescents with acute and chronic pain,*[8] *and for computer*[9] *and tablet-based*[10] *use*."

However, we had not included this for our function outcome. In response, we have now added the following for our description of the KOOS for children:

"*The scale has demonstrated good to excellent psychometric properties in children and adolescents, including reliability and validity*.[11]"

Please note, in making this amendment we realised we had the incorrect reference for the KOOS for children. We have now added the correct reference throughout our manuscript.

Textual considerations:

22. Page 3: Last sentence of introduction: "improves pain, function and other symptoms", shouldn't it be "improves function and reduces pain and other symptoms"? Or do you want to see if minimal footwear results in more pain and other symptoms compared to motion control shoes?

RESPONSE: The reviewer's suggested revision ("…reduces pain….") has the same meaning as our original statement ("improves pain…."), thus we have not made any changes.

23. On the same page at the primary outcomes, for the second primary outcome, a scale has been given, for the first one there has not been one given. However in the article itself there is a scale.

RESPONSE: Thanks for this. We have now added the scale used for our pain primary outcome as follows:

"*The primary outcomes are the 3-month change in i) severity of the worst knee pain experienced over the past week, measured using a numerical rating scale, and ii) the function in sport and play subscale of the Knee Injury and Osteoarthritis Outcome Score for children*."

24. Page 22: There is no clear section of strenghts and limitations of the study in the discussion section.

RESPONSE: The reviewer is correct, however the strengths and limitations have been positioned after the Abstract as per BMJ Open's formatting guidelines for protocol papers:

"Please include a 'Strengths and limitations of this study' section after the abstract. This section should be no more than 5 bullet points relating specifically to the methods - not the results of the study. This will be published as a summary box after the abstract in the final published article." (see here: https://bmjopen.bmj.com/pages/authors#protocol)

25. Looking at the above, I think some revision needs to be done and some addition information is required. I would like to leave it to up to the editor of this journal to decide how to proceed.

RESPONSE: Thank you for taking the time to review our manuscript and for your useful suggestions. We feel these changes have helped to improve the quality and clarity of our manuscript. We hope you find our revisions satisfactory.

Reviewer: 3
This manuscript describes a protocol to evaluate the effectiveness of treating adolescent knee pain and function using minimalist compared to motion-control shoes.

The protocol is very well written, containing a comprehensive description of the planned methods and statistical analyses, and the rationale behind methodological decisions. The methods and analysis plan appear sound and I have no further suggestions or requests for clarification.

RESPONSE: Thank you for the positive feedback.

REFERENCES

1. Paterson KL, Bennell KL, Campbell PK, et al. The effect of flat flexible versus stable supportive shoes on knee osteoarthritis symptoms: a randomized trial. *Annals of internal medicine* 2021;174(4):462-71. doi: 10.7326/M20-6321 [published Online First: 2021/01/12]
2. Sayer TA, Hinman RS, Fortin K, et al. Differences in hip and knee running moments across female pubertal development. *Medicine and science in sports and exercise* 2018;50(5):1015-20. doi: 10.1249/MSS.0000000000001525
3. Crossley KM, Bennell KL, Cowan SM, et al. Analysis of outcome measures for persons with patellofemoral pain: which are reliable and valid? *Archives of Physical Medicine & Rehabilitation* 2004;85(5):815-22.
4. Roos EM, Lohmander LS. The Knee injury and Osteoarthritis Outcome Score (KOOS): From joint injury to osteoarthritis. *Health and Quality of Life Outcomes* 2003;1(1):64.
5. Collins N, Crossley K, Beller E, et al. Foot orthoses and physiotherapy in the treatment of patellofemoral pain syndrome: randomised clinical trial. *BMJ* 2008;337:a1735. doi: 10.1136/bmj.a1735 [published Online First: 2008/10/28]
6. Hossain M, Alexander P, Burls A, et al. Foot orthoses for patellofemoral pain in adults. *Cochrane Database of Systematic Reviews* 2011(1) doi: 10.1002/14651858.CD008402.pub2
7. van der Heijden RA, Lankhorst NE, van Linschoten R, et al. Exercise for treating patellofemoral pain syndrome. *Cochrane Database of Systematic Reviews* 2015(1) doi: 10.1002/14651858.CD010387.pub2
8. Birnie KA, Hundert AS, Lalloo C, et al. Recommendations for selection of self-report pain intensity measures in children and adolescents: a systematic review and quality assessment of measurement properties. *Pain* 2019;160(1):5-18. doi: 10.1097/j.pain.0000000000001377 [published Online First: 2018/09/05]
9. Delgado DA, Lambert BS, Boutris N, et al. Validation of digital visual analog scale pain scoring with a traditional paper-based visual analog scale in adults. *Journal of the American Academy of Orthopaedic Surgeons Global Research & Reviews* 2018;2(3):e088. doi: 10.5435/JAAOSGlobal-D-17-00088
10. Bird M-L, Callisaya ML, Cannell J, et al. Accuracy, validity, and reliability of an electronic visual analog scale for pain on a touch screen tablet in healthy older adults: a clinical trial. *Interact J Med Res* 2016;5(1):e3. doi: 10.2196/ijmr.4910
11. Örtqvist M, Iversen MD, Janarv P-M, et al. Psychometric properties of the Knee injury and Osteoarthritis Outcome Score for Children (KOOS-Child) in children with knee disorders. *British Journal of Sports Medicine* 2014;48(19):1437. doi: 10.1136/bjsports-2013-093164

VERSION 2 - REVIEW

**Reviewer**	**1**
**Name**	**Alonazi, Asma**
**Affiliation**	**Majmaah University, Physical Therapy**
**Date**	**07-Dec-2024**
**COI**	

The manuscript is well-prepared and suitable for publication as a protocol paper, pending minor clarifications.

Suggestions to the author for Improvement:

1. Expand slightly on why minimalist shoes may enhance adherence compared to traditional interventions, emphasizing factors like ease of use, comfort, and appeal to adolescents' preferences.

2. The reliance on self-reported adherence, even with the addition of microsensors, could introduce bias. Explore complementary methods to validate adherence data, such as parental or coach verification or comparison with activity logs.

3. The inability to blind participants fully to their footwear allocation might influence subjective outcomes. Discuss how this will be addressed in data interpretation to minimize potential bias.

4. Consider detailing how the study will control or adjust for confounding variables such as baseline activity levels, previous footwear habits, or other lifestyle factors in the analysis.

5. Since participants are recruited from shared environments like schools or sports clubs, provide more detail on how clustering effects will be handled to ensure robust statistical analyses.

6. The manuscript acknowledges key limitations, including the inability to blind participants and the potential for self-reported adherence bias. Discuss how these limitations will be mitigated during the study or addressed in data interpretation.

7. Expand on the protocols for managing or mitigating adverse events during the trial, including clear reporting and follow-up measures for participants who experience significant discomfort or injury.

8. While the intervention is well-defined, further elaboration on the process of selecting shoe models (e.g., biomechanical properties, adolescent preferences) beyond survey results could strengthen the intervention's credibility.

**Reviewer**	**2**
**Name**	**Koeter, Sander**
**Affiliation**	**Canisius Wilhelmina Hospital, Department of Orthopedics**
**Date**	**17-Dec-2024**
**COI**	

Thank you for your adequate and complete response to our comments. We feel that these alterations have much improved the article and would like to wish you good luck with implementation of this study.

VERSION 2 - AUTHOR RESPONSE

Reviewer: 1

26. Expand slightly on why minimalist shoes may enhance adherence compared to traditional interventions, emphasizing factors like ease of use, comfort, and appeal to adolescents' preferences.

RESPONSE: We have added the following sentence:

"*Hence, footwear is an inexpensive, easy-to-use approach that may appeal to adolescent's preferences."*

Please note we did not include comfort given we are unsure if both intervention shoes will be perceived as comfortable by participants (note: comfort is an "other measure").

27. The reliance on self-reported adherence, even with the addition of microsensors, could introduce bias. Explore complementary methods to validate adherence data, such as parental or coach verification or comparison with activity logs.

RESPONSE: Thank you for this suggestion. As our trial has been registered, and participant recruitment has commenced and is ongoing, we are unable to add any additional measures. We feel our self-reported measure of adherence, as well as use of sensors to measure wear time, is adequate. Nonetheless, we have acknowledged the limitation of self-reported adherence (and the strength of using a microsensor) in the "Strengths and limitations of this study" section of our manuscript.

28. The inability to blind participants fully to their footwear allocation might influence subjective outcomes. Discuss how this will be addressed in data interpretation to minimize potential bias.

RESPONSE: The inability to blind participants to their own treatment (footwear allocation in this trial) is common to all similar randomised controlled trials, as it is not possible to blind participants receiving an active treatment/intervention. In our trial, participants are blinded to the alternate footwear group and study hypotheses, via limited disclosure. Participants are only informed that the trial compares two footwear styles that have the potential to improve their kneecap symptoms, and will be blinded to the alternate condition. We have acknowledged this as a limitation, and will also do this when interpreting our study findings in the main paper. Although we described our blinding process in our Methods section, we have added the following in an attempt to make it clearer:

"Participants/guardians are not informed about the shoe 'class' (i.e., minimalist or motion control) or models provided to the comparison group participants, and are only told that the trial compares two footwear styles that have the potential to improve their kneecap symptoms."

OBJECTIVES

29. Consider detailing how the study will control or adjust for confounding variables such as baseline activity levels, previous footwear habits, or other lifestyle factors in the analysis.

RESPONSE: Given that this is a randomised controlled trial, we expect the randomisation process to produce balanced groups at baseline for potential prognostic factors such as baseline activity levels, previous footwear habits, and other lifestyle factors. This will minimise any issues related to confounding. Primary analyses will be conducted without adjustment for these variables, assuming the randomisation process has ensured the required balance. During the development of the detailed statistical analysis plan, we will outline any key prognostic factors which we would be particularly concerned about treatment imbalances and propose considering these as adjustment variables in sensitivity analyses.

30. Since participants are recruited from shared environments like schools or sports clubs, provide more detail on how clustering effects will be handled to ensure robust statistical analyses.

RESPONSE: This is an individually randomised trial, where participants are randomly allocated to treatment groups independent of their recruitment site or any shared environment. Unlike a cluster randomised trial, where entire clusters (e.g., schools or sports clubs) are randomised together, the individual randomisation protects against any potential clustering effects from the recruitment strategy. With individual randomisation, participants within the same cluster (e.g., from the same school) have an equal probability of being allocated to either treatment arm. This ensures that any potential clustering effects are balanced across the treatment groups, and therefore the primary analyses will be undertaken without adjustment for clustering

31. The manuscript acknowledges key limitations, including the inability to blind participants and the potential for self-reported adherence bias. Discuss how these limitations will be mitigated during the study or addressed in data interpretation.

RESPONSE: Please see our response to comment #4.

32. Expand on the protocols for managing or mitigating adverse events during the trial, including clear reporting and follow-up measures for participants who experience significant discomfort or injury.

RESPONSE: This trial is testing commercially available footwear that is purchased and worn by people in the course of their usual lifestyle. Thus, adverse events from the allocated footwear interventions are unlikely. If adverse events do occur, they are likely to be minor and transient, consistent with our previous trial evaluating similar footwear for knee OA.[1] Whilst we have explained our adverse event process, we have further elaborated as follows:

"Participants will also be advised that if they experience an adverse event, they can contact the study researchers at any time. A member of the research team (KP, a study podiatrist) will manage any adverse events reported by participants."

33. While the intervention is well-defined, further elaboration on the process of selecting shoe models (e.g., biomechanical properties, adolescent preferences) beyond survey results could strengthen the intervention's credibility.

RESPONSE: Thank you for the comment. For clarity, there were no other processes for selecting the study shoe models other than those we outlined. As we explain in the "Footwear intervention" section of our Methods (which covers two pages), the shoe properties were based on those showing minimalist shoe led to lower patellofemoral joint contact force and stress during a brief bout of running, compared to motion control shoes (Table 1). It also explains that the two study shoes were

selected based on adolescent preferences from our survey, which included a range of shoes that had the properties listed in Table 1.

Reviewer: 2
Thank you for your adequate and complete response to our comments. We feel that these alterations have much improved the article and would like to wish you good luck with implementation of this study.

RESPONSE: Thank you for this positive feedback, and for your helpful review.

REFERENCES

1. Paterson KL, Bennell KL, Campbell PK, et al. The effect of flat flexible versus stable supportive shoes on knee osteoarthritis symptoms: a randomized trial. *Annals of internal medicine* 2021;174(4):462-71. doi: 10.7326/M20-6321 [published Online First: 2021/01/12]
